# Angiosarcoma with Synchronous Cutaneous and Small Bowel Involvement: A Report of a Rare Presentation

**DOI:** 10.7759/cureus.3650

**Published:** 2018-11-28

**Authors:** Erin Reis, Kadra Kalamaha, Hongchen Jia, Hermina D Fernandes

**Affiliations:** 1 Internal Medicine, University of North Dakota School of Medicine and Health Sciences, Bismarck, USA; 2 Pathology, Sanford Health, Bismarck, USA

**Keywords:** angiosarcoma, cutaneous angiosarcoma, metastatic cancer, cirrhosis, gastric antral vascular ectasia, radiation, paclitaxel, bevacizumab

## Abstract

Angiosarcomas are mesenchymal neoplasms of vascular origin that represent approximately 2% of soft tissue sarcomas. We discuss the case of a 75-year-old female who had presented with a purple nodular rash along the bilateral nasolabial folds. Upon further work-up, she was diagnosed with angiosarcoma, with the confirmed involvement of multi-focal sites. These included biopsy proven sites of the face and duodenum along with the radiographic involvement of the lungs, liver, and osseous tissue. We report this unique presentation of a rare malignancy and the treatment course with radiation, paclitaxel, and bevacizumab. We also discuss the implications of her co-morbid liver cirrhosis and gastric antral vascular ectasia (GAVE) in terms of its influence on the development of the angiosarcoma and treatment response.

## Introduction

Angiosarcomas are rare mesenchymal neoplasms of endothelial cells which fall under the umbrella of soft tissue sarcomas. The annual incidence of soft tissue sarcomas in the United States is estimated to be 10,000 cases per year [1]. Specifically, angiosarcomas are estimated to comprise about 1-2% of these soft tissue sarcoma cases [2]. Angiosarcomas can arise anywhere in the body, with about 60% of cases initially presenting in the skin as a cutaneous angiosarcoma [1]. The head and neck are the most common sites of cutaneous angiosarcoma, contributing 59.3% in a large incidence analysis [2]. A number of risk factors for angiosarcoma have been elucidated in the literature including, but not limited to, radiation therapy, chronic lymphedema, environmental exposures (vinyl chloride, thorium dioxide, ultraviolet light, arsenic, anabolic steroids, foreign bodies), benign vascular lesions, arteriovenous fistulas, xeroderma pigmentosum, familial syndromes, retinoblastoma, and previous primary malignancy [3-5].

Here, we report a unique case of metastatic angiosarcoma with the involvement of the skin and small bowel, and have discussed the implications of comorbidities including cirrhosis and gastric antral vascular ectasia (GAVE).

## Case presentation

**Figure 1:**
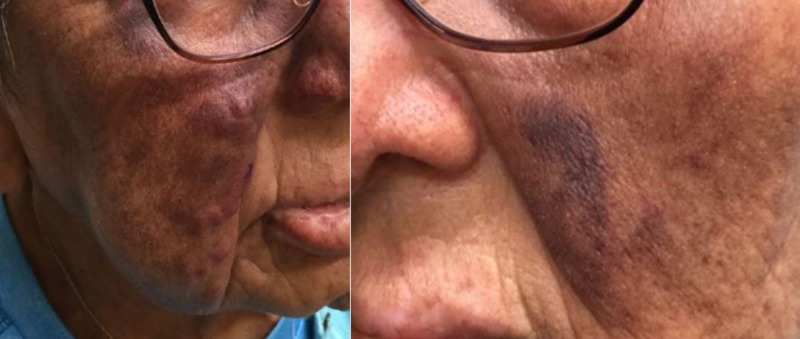
Facial Lesion 'Port wine’ purple nodular rash on the patient's nasolabial folds

A 75-year-old Native American female presented to dermatology with a ‘port wine’ purple nodular rash on her nasolabial folds, of 12 months duration (Figure 1). There was no associated pruritus, burning, pain, or bleeding from the area. She did report drainage of some clear fluid when pressure was applied to the area. On physical exam, there were areas of raised, papular, nodular purple growth along the bilateral nasolabial folds. There was no evidence of drainage or infection. There were no oral lesions or skin lesions elsewhere on her body upon complete dermatological exam. Her medical history was remarkable for cirrhosis, deemed cryptogenic or secondary to non-alcoholic steatohepatitis (NASH) following evaluation by gastroenterology. Her medical history also included iron deficiency anemia secondary to GAVE, type II diabetes mellitus, hypertension, asthma, and endometrioid carcinoma of the ovary.

**Figure 2:**
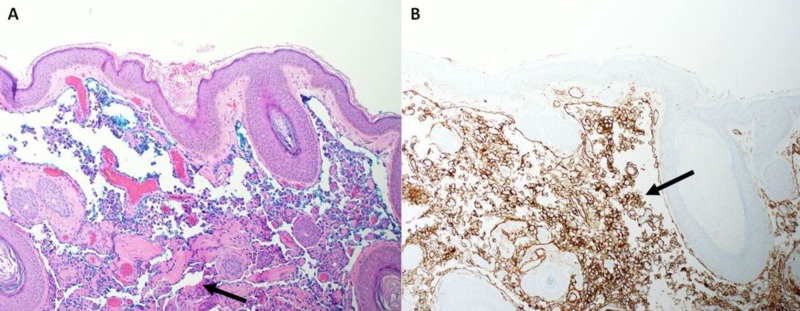
Skin Biopsy A. Vasoformative growth, with anastomosing channels in upper dermis (H&E, x10). B. Tumor cells on the formalin-fixed section demonstrating strong membrane stain for CD31 (immunohistochemistry, x10).

A 3-mm punch biopsy of the right nasolabial fold lesion demonstrated an atypical vascular lesion extending to the tissue margins. Sections revealed prominent vascular dilatation with papillary fragments and associated endothelial proliferation with cytologic atypia (Figure 2). A cluster of differentiation (CD)31 stain highlighted lesional cells, representing angiosarcoma. 

**Figure 3:**
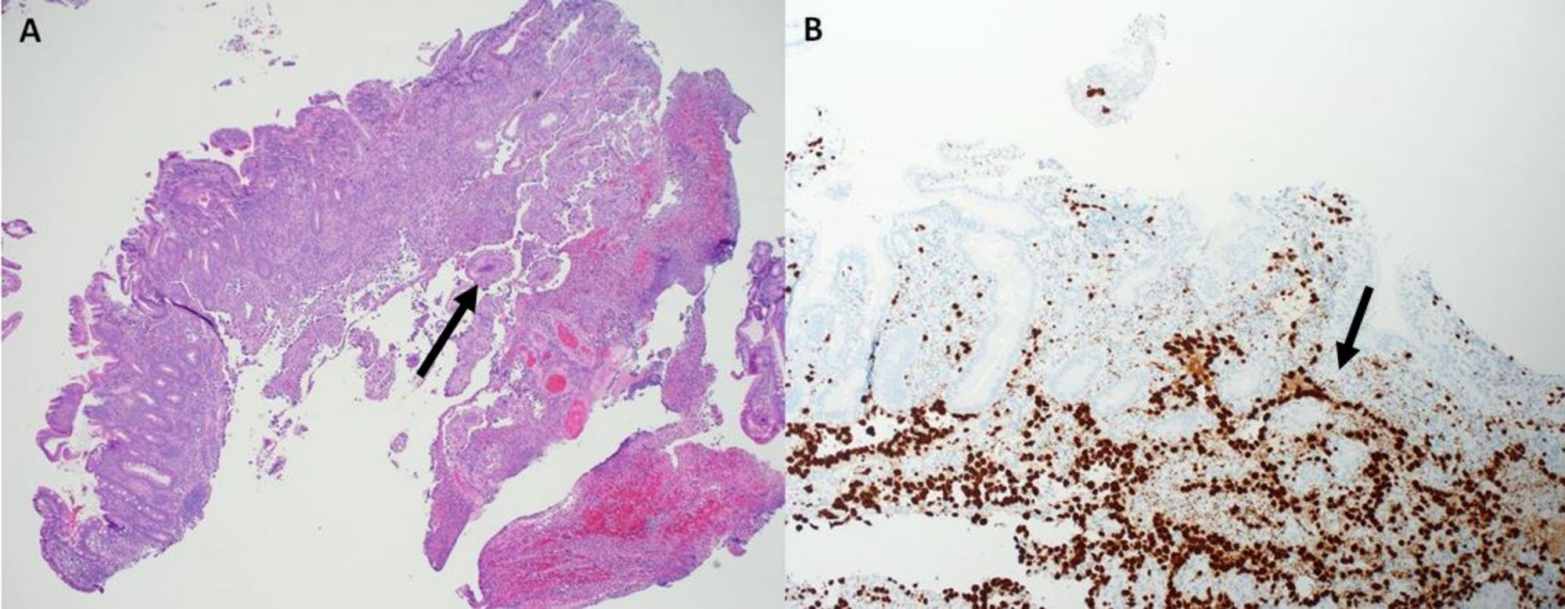
Duodenal Biopsy A. Anastomosing vessels in lamina propria (H&E, x4). B. Tumor cells of angiosarcoma demonstrating strong nuclear stain for ERG (immunohistochemistry, x4).

She also underwent a surveillance gastrointestinal endoscopy due to her history of cirrhosis, and a duodenal ulcer was incidentally discovered. A biopsy was performed that revealed duodenal mucosa with ulceration and granulation tissue along with atypical, neoplastic proliferation of cells growing in sheets (Figure 3). Immunostains for erythroblast transformation-specific (ETS)-related gene (ERG) and friend leukemia integration 1 transcription factor (FLI1) were positive, confirming endothelial differentiation and thus consistent with angiosarcoma involving the duodenum.

**Figure 4:**
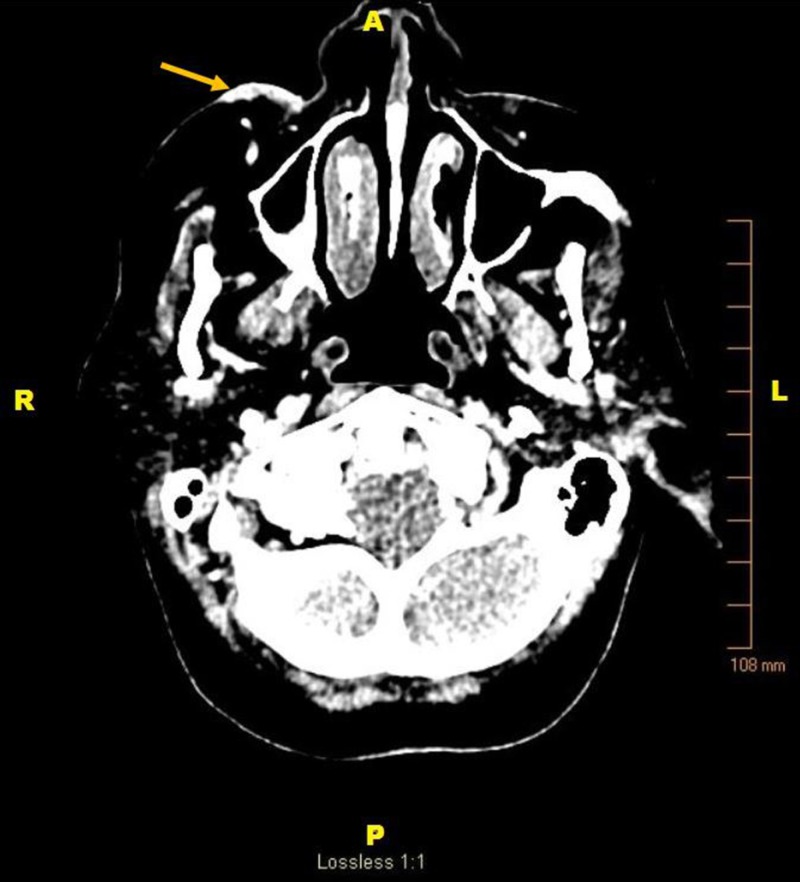
Computed Tomography Head Computed tomography (CT) head with contrast pre-treatment showing (*yellow arrow*) cutaneous thickening and irregularity throughout the right face compatible with angiosarcoma. Legend: A - anterior; P - posterior; R -right; L - left

**Figure 5:**
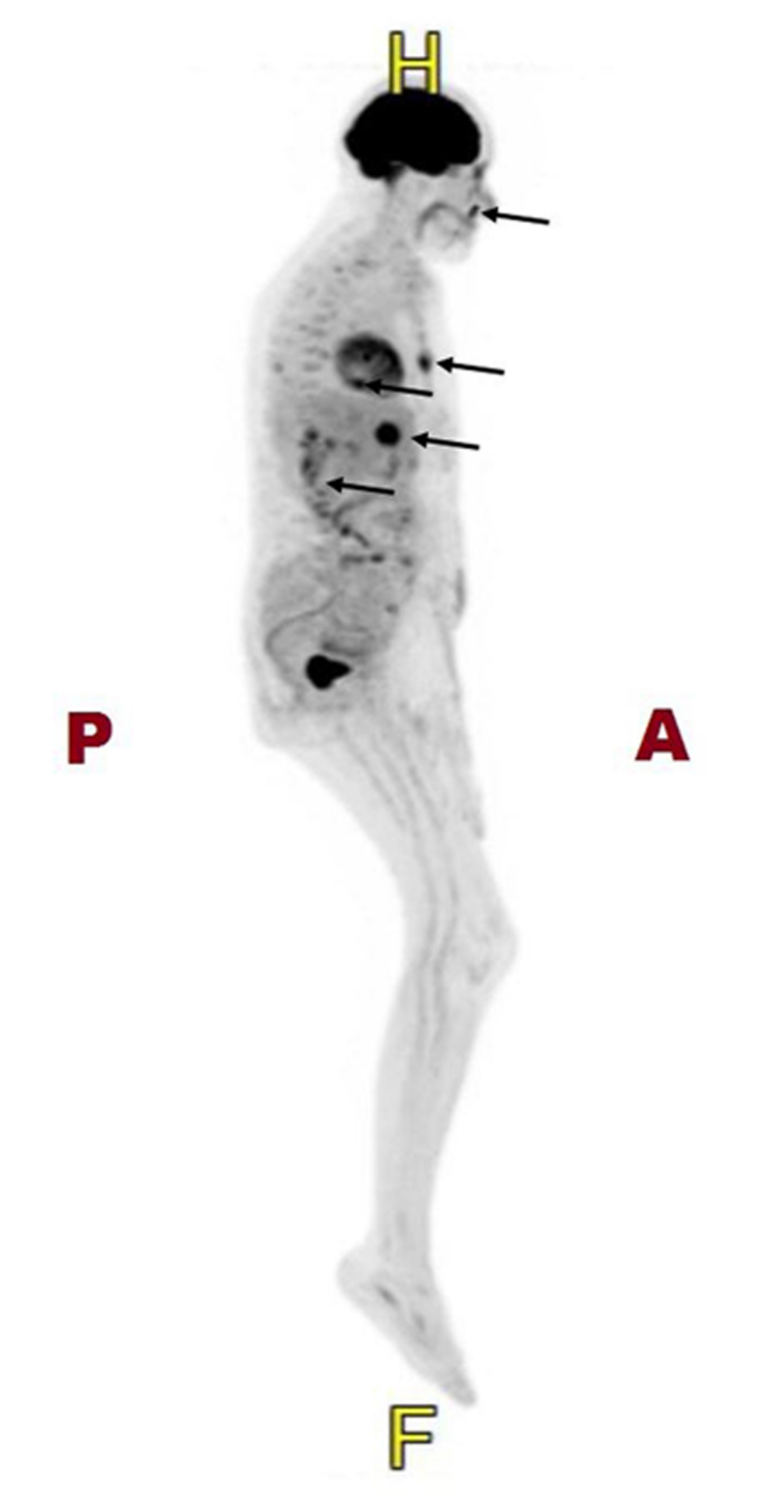
PET scan PET scan pre-treatment showing increased uptake at sites of facial, sternal, pulmonary, liver and spinal lesions (*arrows*). Legend: A, anterior; P, posterior; H, head; F, foot PET: Positron emission tomography

Staging evaluation was performed. Computed tomography (CT) imaging demonstrated right face superficial angiosarcoma without the invasion of deep tissues (Figure 4), bilateral lower lung nodules indeterminate for malignancy, and a 1.1 cm hypo-attenuated lesion of the right liver lobe indeterminate for malignancy. Positron emission tomography (PET) CT demonstrated hyper-metabolic activity in the face, consistent with the known lesion, and additionally hypermetabolic activity in the right scapular spine, distal sternum, pulmonary nodules of the left lung, right lobe of the liver, and T12 vertebral body (Figure 5). These findings suggested osseous and soft tissue metastases. A single liver lesion was biopsied and demonstrated changes consistent with chronic hepatitis and cirrhosis with no evidence of malignancy.

These findings confirmed the angiosarcoma of the face with multiple synchronous sites, including the duodenum. The patient was presented at the multidisciplinary tumor board where it was recommended that she receive palliative radiation for local control of the lesion on the face/nasolabial folds. She was also started on systemic therapy with intravenous paclitaxel as per the Phase II Trial of Weekly Paclitaxel for Unresectable Angiosarcoma (ANGIOTAX) study [6].

She experienced some treatment delay in between chemotherapy cycles due to radiation-induced myelosuppression. Side effects were monitored closely, given her history of cirrhosis and baseline bicytopenia. She also experienced grade 2 cutaneous toxicity from radiation therapy including painful erythematous lesions in the mouth and over the lips as well as blepharitis. Post-radiation skin changes on the face eventually healed well, following completion of radiation. She did not experience any paresthesia with paclitaxel.

**Figure 6:**
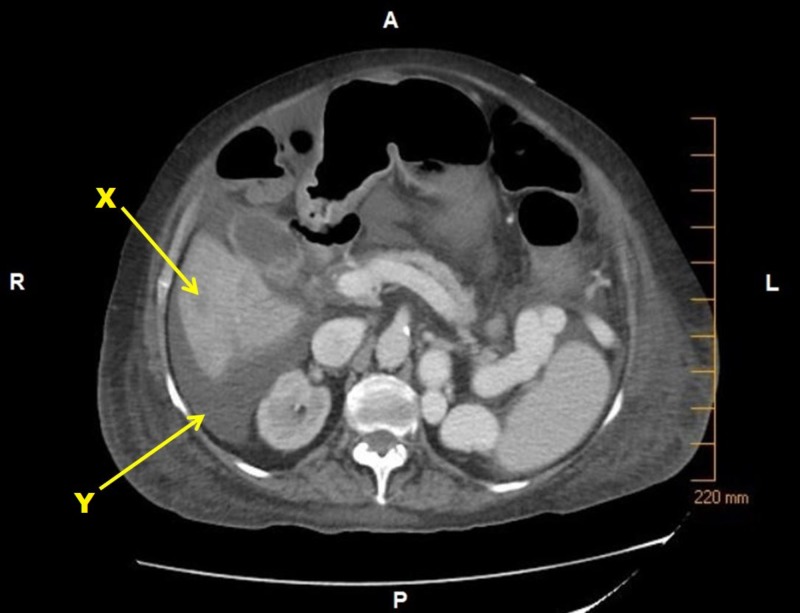
Computed Tomography (CT) Abdomen Computed tomography (CT) abdomen with contrast post-treatment with first-line paclitaxel showing X. Ill-defined low-attenuating lesion in the inferior right hepatic lobe laterally measuring up to 2.0 x 2.1 cm concerning for metastasis Y. Ascites. Legend: A - anterior; P - posterior; R - right; L - left

Imaging following two months of systemic chemotherapy revealed interval progression of hepatic and vertebral metastases (Figure 6).

Due to disease progression, paclitaxel was discontinued and treatment was changed to second-line bevacizumab based on the available data from a phase II trial [7]. Following cycle 1 of bevacizumab, she experienced decompensated cirrhosis along with spontaneous bacterial peritonitis and ascites. Treatment was deferred due to multiple hospitalizations in the next few weeks due to decompensated cirrhosis. The patient and her family then elected to transition to hospice due to worsening quality of life. She died six months following her diagnosis of angiosarcoma with complications from decompensated liver cirrhosis.

## Discussion

Angiosarcomas are tumors arising in either the blood vessels or lymphatics. The scalp and face are the most common sites of origin [8]. They are aggressive in nature, have a high metastatic potential, and are associated with poor prognosis. A large meta-analysis found the five-year survival rate for angiosarcomas of the scalp and face to be 33.5% [9]. It has been estimated that 16-46% of angiosarcoma cases present with metastatic disease or as multi-focal lesions, with hematogenous spread to lung and brain to be most common [1,9]. Factors associated with better prognosis for cutaneous angiosarcomas include having an age lower than 70 years, a lower number of affected regions, lack of metastatic disease, involvement of the face versus the scalp, negative margins upon resection, tumor size less than 5 cm, histologic growth demonstrating greater than 80% solid areas, and programmed death-ligand 1 (PD-L1) expression [8-11].

The involvement of the small bowel is even more uncommon and is associated with worse outcomes as compared to cutaneous angiosarcomas. A recent literature review of previously published data examining small bowel angiosarcomas concluded that survival with intestinal involvement is less than one year and confers an extremely poor prognosis [12]. A retrospective analysis of 66 patients with small intestine angiosarcomas demonstrated a mean survival time of 30 weeks from diagnosis, with death usually associated with uncontrollable gastrointestinal bleeding or respiratory failure [13].

In our patient, due to synchronous presentation, it is not possible to determine whether the cutaneous lesion or the duodenal lesion was the primary tumor. A retrospective review of the published literature suggests that a majority of small bowel angiosarcomas are primary tumors. It was also noted that the likelihood of metastases to the intestine was the highest when the primary sites of the tumor were the skin or aorta [13]. The presence of epithelioid variant cells in the cutaneous biopsy from the face, as in our case, is more likely to be associated with angiosarcoma of the soft tissues as opposed to an intestinal primary angiosarcoma [3]. The tendency of these tumors to be multifocal, with an ability to develop at any site and a variety of presentations, make it challenging to ascertain the primary site of the tumor, as in this case.

Some data have suggested that the benign vascular lesions may be a risk factor for malignant transformation into angiosarcoma [4]. In this case, the patient’s history of GAVE could be implicated in the development of angiosarcoma, although these could also be related to portal hypertension from her cirrhosis.

Our patient also had cirrhosis deemed secondary to NASH or cryptogenic cirrhosis upon prior investigation. Exposure to vinyl chloride has been implicated as a risk factor for hepatic angiosarcoma and cirrhosis [14-15]. It is reasonable to consider the possibility of angiosarcoma arising in the liver and coexisting in the context of cirrhosis. This incidence of hepatic angiosarcoma coexisting in the background of cirrhosis is especially high when both are linked to vinyl chloride exposure [14]. It is unclear whether the patient had a prior history of vinyl chloride exposure. She was also found to have hepatic tumor deposits radiographically during the course of her disease, which raises the possibility of a hepatic source of the tumor.

In the context of treatment, complete excision with wide margins is the treatment of choice for locally invasive disease. However, cutaneous angiosarcomas have been associated with high rates of local recurrence [11]. Due to the propensity for local infiltration, radiation therapy is also utilized to achieve local disease control [7]. Metastatic angiosarcomas have been proven to be sensitive to paclitaxel, with the median progression-free survival of 5  to 7.6 months [16]. Weekly and every three-week schedules have also been described with varying success [16]. Molecular targeted therapies such as sorafenib have been reported to delay the progression of the disease with a progression-free survival of 31% at six months [16]. Imatinib has also been reported to show a modest benefit with a progression-free survival benefit of 20% at three months [16]. Another phase II study demonstrated that anti-angiogenic agents like bevacizumab had activity against tumor with a median time to progression of 26 weeks [7].

## Conclusions

Though the incidence is low, their aggressive behavior and poor prognosis make angiosarcomas a threatening diagnosis that should be identified and treated as soon as possible. The risk factors that should alert a clinician to look for an angiosarcoma are many. They include radiation therapy, chronic lymphedema, environmental exposures, ultraviolet light, arsenic, anabolic steroids, foreign bodies, benign vascular lesions, and arteriovenous fistulas. This malignancy is also commonly associated with xeroderma pigmentosum, familial syndromes, retinoblastoma, and previous primary malignancy. The prognosis for a metastatic disease remains in evolution with the availability of multiple agents for targeted therapy such as imatinib, sorafenib, and bevacizumab.
